# Inhibition of GSK3β Reduces Ectopic Lipid Accumulation and Induces Autophagy by The AMPK Pathway in Goat Muscle Satellite Cells

**DOI:** 10.3390/cells8111378

**Published:** 2019-11-01

**Authors:** Linjie Wang, Xin Liu, Siyuan Zhan, Jiazhong Guo, Shizhong Yang, Tao Zhong, Li Li, Hongping Zhang, Yan Wang

**Affiliations:** 1 Farm Animal Genetic Resources Exploration and Innovation Key Laboratory of Sichuan Province, College of Animal Science and Technology, Sichuan Agricultural University, Chengdu 611130, Sichuan, China; wanglinjie@sicau.edu.cn (L.W.); frankielx@163.com (X.L.); siyuanzhan@sicau.edu.cn (S.Z.); jiazhong.guo@sicau.edu.cn (J.G.); zhongtao@sicau.edu.cn (T.Z.); lily@sicau.edu.cn (L.L.); zhp@sicau.edu.cn (H.Z.); 2 Institute of Liangshan Animal Husbandry and Veterinary Science, Xichang 615042, Sichuan, China; xcysz1565@163.com

**Keywords:** GSK3β, muscle satellite cells, lipid accumulation, AMPK, autophagy

## Abstract

Ectopic lipid accumulation in muscle is important not only for obesity and myopathy treatment, but also for meat quality improvement in farm animals. However, the molecular mechanisms involved in lipid metabolism in muscle satellite cells are still elusive. In this study, SB216763 reduced GSK3β activation by increasing the level of pGSK3β (Ser9) and decreasing the level of total GSK3β protein. GSK3β inhibition decreased lipid accumulation and downregulated the expression level of lipogenesis-related genes in the adipogenic differentiation of goat muscle satellite cells. Furthermore, SB216763 treatment increased the levels of pAMPKα (T172) and pACC (Ser79). Further, we found that GSK3β inhibition promoted levels of LC3B-II and reduced the protein levels of p62 to induce the autophagy in muscle satellite cells. Taken together, our results provide new insight into a critical function for GSK3β: modulating lipid accumulation in goat muscle satellite cells through activating the AMPK pathway.

## 1. Introduction

Muscle satellite cells reside beneath the basal lamina and are responsible for postnatal muscle growth and regeneration [1]. Muscle satellite cells are multipotential stem cells that can transdifferentiate into adipocytes, osteoblasts and myotubes [2]. Previous studies have demonstrated that several signaling pathways are involved in the transition from myogenesis to adipogenesis. For example, PRDM16 (PR domain containing 16) interacts with C/EBP-β (CCAAT enhancer binding protein β) and then controls the transdifferentiation from myoblast to brown adipocyte [3]. In addition, miR-133 targets the *PRDM16* gene and prevents satellite cells from differentiating into brown adipocytes [4]. Liver kinase B1 (Lkb1) deletion in myoblasts promotes the lipid accumulation and the expression of lipid metabolism related genes through activating the AMPK (AMP-activated protein kinase) pathway [5]. There is more lipid accumulation in skeletal muscle of Wnt10bknockout mice compared to WT mice and Wnt10b deletion promotes adipogenic differentiation in myoblasts [6]. However, the molecular mechanisms involved in lipid metabolism in muscle satellite cells are still elusive.

GSK3β (glycogen synthase kinase 3β) is a serine/threonine protein kinase, which has been related to various cellular processes, including diabetes, inflammation, aging, embryonic development and muscle regeneration [7,8]. A GSK3β global knockout in mice is embryonically lethal, and is caused by severe liver degeneration [9]. Skeletal muscle-specific GSK3β knockout mice have improved glucose tolerance and enhanced insulin-stimulated glycogen deposition [10]. In addition, skeletal muscle-specific GSK3β deletion prevents muscle atrophy though increasing muscle mass and muscle protein synthesis [11]. In differentiated C2C12 cells, the inactivation of GSK3β promotes myotube fusion, muscle creatine kinase (MCK) activity, and the expression of muscle-specific genes [12,13]. SB216763 is an ATP-competitive inhibitor of GSK3β, which is a widely used to inhibit GSK3β kinase activity [14]. In addition, the inhibition of GSK3β by SB216763 results in the increased phosphorylation of pGSK3β (Ser9), a negatively regulated phosphorylation site of GSK3β kinase activity [15]. Our previous study demonstrated that GSK3β inhibition with SB216763 induced the myogenic differentiation and increased the expression level of *MyHC2a* (myosin heavy chain 2a) by transcription factor NFATc2 (nuclear factor of activated T-cells, cytoplasmic 2) in goat muscle satellite cells [16]. Although previous studies have demonstrated that GSK3β plays an important role in skeletal muscle development, the function of GSK3β in lipid accumulation of skeletal muscle satellite cells is completely unknown. 

In humans, skeletal muscle wasting diseases, such as Duchenne muscular dystrophy, are associated with increased ectopic lipid accumulation [17]. In addition, aging in skeletal muscle is characterized not only by decreased muscle integrity but also by increased ectopic lipid accumulation [18]. In farm animals, the intramuscular fat content has an important role on meat quality traits, including flavor, juiciness and tenderness [19]. Therefore, understanding the molecular mechanism of ectopic lipid accumulation in skeletal muscle is important not only for meat quality improvement, but also for obesity and myopathy treatment. In this study, GSK3β inhibition decreased lipid accumulation through AMPK in muscle satellite cells. Furthermore, GSK3β inhibition promoted levels of LC3B-II (microtubule-associated protein 1 light chain 3B) and reduced the protein levels of p62 (sequestosome 1) to induce the autophagy in muscle satellite cells.

## 2. Materials and Methods

### 2.1. Ethics Statement

All research involving animals was conducted according to the approved protocols of the Institutional Animal Care and Use Committee at the College of Animal Science and Technology, Sichuan Agricultural University, Sichuan, China, under permit number DKYB20110807.

### 2.2. Muscle Satellite Cells’ Isolation and Adipogenic Differentiation

The pregnant Chuanzhong black ewes were raised at the breeding center of the Sichuan Agricultural University, Ya’an, China. These ewes were fed a standard diet (forage to concentrate ratio, 70:30) twice per day at 07:00–09:00 and 16:00–18:00, and drank water ad libitum. Ultimately, the skeletal muscle samples were collected from Chuanzhong black goats 3 days after birth. 

Muscle satellite cells were isolated using a method previously described [20]. In brief, the skeletal muscles were digested with 0.2% pronase (Sigma, MO, USA) at 37 °C. Cell suspensions were filtrated through 200 μm and 40 μm Nytex filters, respectively; then, centrifuged at 800× *g* for 10 min. Finally, the cells were plated in growth medium containing DMEM with 15% FBS (Gibco, CA, USA) and 1% antibiotics at 37 °C with 5% CO_2_. For adipogenic differentiation, the satellite cells reached full confluence, and were then induced with medium containing DMEM, 15% FBS, 10 μg/mL insulin, 1 μM dexamethasone and 0.5 mM 3-isobutyl-1-methylanxthine (IBMX) for 4 days. Next, they were induced in medium containing DMEM, 15% FBS and 10 μg/mL insulin for 3 days. To evaluate the effect of GSK3β in lipid accumulation of muscle satellite cells, cells were treated with 10 μM SB216763 for 0, 4 and 7 days, respectively. To determine whether GSK3β regulates ectopic lipid accumulation through the AMPK pathway, cells were treatment with 10 μM SB216763 in the presence or absence of 2 μM dorsomorphin (Compound C) (Selleck, TX, USA) for 4 days. 

### 2.3. Immunofluorescence Assa

Expression of Pax7 in muscle satellite cells was detected by Immunofluorescence. Briefly, the cells were fixed in 4% paraformaldehyde for 15 min at 37 °C and washed three times with PBS. The cells were then permeated with 0.5% Triton X-100 for 10 min, which was followed by blocking with 2% bovine serum albumin (BSA) for 60 min. The cells were then incubated with primary antibody (rabbit anti-Pax7, Absin, Shanghai, China) overnight at 4 °C, then incubated with fluorochrome-labeled secondary antibody (goat anti-Rabbit IgG-AlexaFluor 488, Absin, Shanghai, China) for 2 hours at 37 °C. Finally, they were washed in PBS and stained with DAPI for 45 min at room temperature. Fluorescent images were captured using a fluorescence microscope (Nikon, Tokyo, Japan). The percentage of Pax7 positive cells was estimated from six randomly chosen fields and expressed as the number of Pax7 positive cells divided by the total number of nuclei in the same field.

### 2.4. Cell Viability Assay

The cell counting kit-8 (CCK-8) assay was used to detect cell viability. Cells were seeded into a 96-well plate in growth medium containing 10 μM SB216763 for 0, 4 and 7 days, respectively. Briefly, CCK-8 solution (10 μL/well) was then added and incubated for 2 hours at 37 °C. The cell viability was assessed by the absorbance at 450 nm using a microplate reader (Thermo Fisher, DE, USA). Quantitative values are expressed as percentages of values from cells treated with SB216763 compared with the same treatment times in untreated control cells. 

### 2.5. Caspase-3 and Caspase-8 Activity Assay 

The activity of caspase-3 and caspase-8 was measured using the caspase-3 and caspase-8 activity kit (Beyotime, Haimen, China) according to the manufacturer’s instructions. Cells were seeded into a 96-well plate in growth medium containing 10 μM SB216763 for 0, 4 and 7 days, respectively. Assays were performed on 96-well plates by incubating cell lysates in reaction buffer containing caspase-3 substrate (Ac-DEVD-pNA) and caspase-8 substrate (Ac-IETD-pNA), respectively. Then, cell lysates were centrifuged at 16,000× *g* for 10 min at 4 °C. The supernatant samples were assessed by absorbance at 405 nm using a microplate reader (Thermo Fisher, DE, USA). Quantitative values are expressed as percentages of values from cells treated with SB216763 compared with the same treatment times in untreated control cells.

### 2.6. Oil Red O (ORO) Staining

Adipocytes were washed with PBS and fixed with 4% formaldehyde at room temperature for 1 h. Then, ORO working solution was added to each well for 30 min. Finally, the cells were washed with 60% isopropanol to remove excess ORO, and pictured. 

### 2.7. Triglyceride (TG) Content Assay

Triglyceride content was quantified using a triglyceride assay kit (Applygen, Beijing, China). Briefly, adipocytes were treated with cell lysis buffer, and then, the supernatant was collected. The quantification of triglyceride was normalized to the cellular protein concentration using a BCA protein assay kit (Applygen, Beijing, China). Absorbance was measured at a 550 nm wave length on a microplate reader (Thermo Fisher, DE, USA). 

### 2.8. Total RNA Isolation, cDNA Synthesis and Qpcr Analysis

The total RNA was extracted using Trizol reagent (Invitrogen, USA). The NanoDrop 2000 spectrophotometer (Thermo Fisher, DE, USA) was used to determine the RNA purity and quantity. Then 2 μg of total RNA was reversed transcribed into cDNA using the PrimeScript™ RT reagent Kit (Takara, Tokyo, Japan). The qPCR was carried out with SYBR Green Master Mix (Takara, Japan), cDNA template and specific primer pairs (Table S1) using a Bio-Rad CFX96 qPCR instrument (Bio-Rad, California, CA, USA). The target gene expression relative to *PPIA* gene was analyzed by the Ct (2^−△△Ct^) method. Comparisons were made by one-way ANOVA in SAS software version 8.01, and Duncan’s new multiple range tests were used to analyze statistical significance.

### 2.9. Western Blotting

Total protein was isolated from muscle satellite cells using a Tissue Total Protein Extraction Kit (Sangon, Shanghai, China) and concentrations were determined using a BCA Protein Assay Kit (Sangon, Shanghai, China). Then, proteins were separated in 10% polyacrylamide gel, moved to polyvinylidene difluoride (PVDF), blocked with 2% BSA for 60 min at room temperature, and then, incubated with primary antibodies overnight at 4 °C. The ACC, pACC (Ser79), GSK3β and pGSK3β (Ser9) antibodies were from Cell Signaling Technology (Danvers, MA, USA). AMPK alpha, pAMPK alpha (Thr172) and LC3B-II antibodies were from Affinity Biosciences (Cincinnati, OH, USA). HRP-goat anti Rabbit, GAPDH and p62 antibodies were from Abcam (Cambridge, UK). Immunoblots were performed over two hours at room temperature using HRP-labeled goat anti-rabbit IgG, followed by detection using an ECL detection system (Beyotime, Shanghai, China). 

## 3. Results

### 3.1. Isolation and Identification of Goat Muscle Satellite Cells

**Figure 1:**
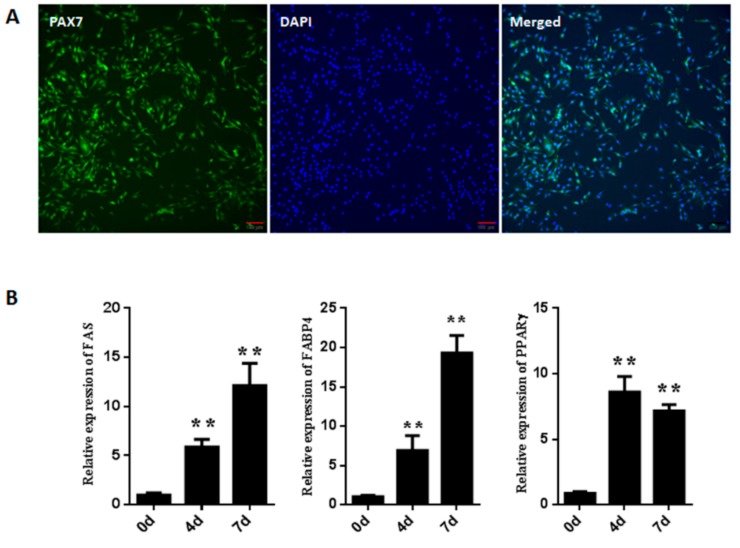
The purity and adipogenic differentiation of goat muscle satellite cells. (**A**) Immunofluorescence analysis of Pax7 in goat muscle satellite cells. Cells were stained with PAX7 antibody and nuclei were stained with DAPI. Scale bars: 100 μm. (**B**) Relative expressions of adipogenic-regulated genes (*FABP4*, *FAS* and *PPARγ*) during cell differentiation at 0, 4 and 7 days by qPCR. Error bars represent SEM of three separate experiments. ** *p* < 0.01 relative to 0 d level.

Pax7 (paired box protein 7), a muscle satellite cell marker, was used to evaluate the purity of the positive muscle satellite cells. As shown in Figure 1A, the percentage of Pax7 positive cells was 97.6% + 0.7%, indicating that most cells were muscle satellite cells and a high purity of satellite cells could be obtained using this method. To determine whether the muscle satellite cells were adipogenic, the cells were induced in adipogenic differentiation medium for 0, 4 and 7 days. Then, the expression level of adipogenic-related genes was determined in three stages respectively. As shown in Figure 1B, the genes’ (*FABP4*, *FAS* and *PPARγ*) expression levels were significantly (*p* < 0.01) up-regulated during the course of the adipogenic differentiation, reaching their highest expression levels at 7 days, indicating that muscle satellite cells can be differentiated into adipocytes by adipogenic differentiation medium. 

### 3.2. Cytotoxicity and Stress Effects of Sb216763 on Muscle Satellite Cells

**Figure 2:**
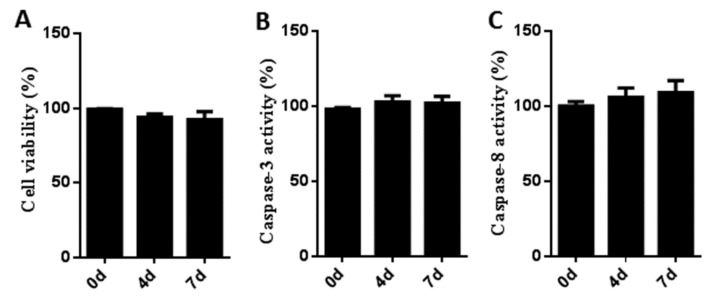
Cytotoxicity and stress effects of SB216763 on muscle satellite cells (**A**) The cytotoxicity of SB216763 on muscle satellite cells was measured by CCK-8 assay. Cells were seeded into a 96-well plate in growth medium with or without 10 μM SB216763 for 0, 4 and 7 days, respectively. Error bars represent the SEMs of three separate experiments. (**B**,**C**) The activities of caspase-3 and caspase-8 were measured by the absorbance at 405 nm using a microplate reader (Thermo Fisher, DE, USA). Error bars represent the SEMs of three separate experiments. Quantitative values are expressed as percentages of values from cells treated with SB216763 compared with the same treatment times in untreated control cells.

To measure the cytotoxicity of SB216763 on muscle satellite cells, the CCK-8 assay was employed. Cells were cultured in growth medium with or without SB216763 (10 μM) for 0, 4 and 7 days. As shown in the Figure 2A, there are no significant differences among the three groups, indicating that the concentration of 10 μM of SB216763 did not affect the viability of muscle satellite cells. To determine whether satellite cells were under stress conditions after SB216763 (10 μM) treatment, the activities of caspase-3 and caspase-8 were evaluated. As shown in the Figure 2B,C, the activities of caspase-3 and caspase-8 showed no significant differences when the satellite cells were exposed to SB216763 for 0, 4 and 7 days compared with the same treatment times in untreated control cells.

### 3.3. SB216763 Affects the Levels of Pgsk3β (Ser9) and Gsk3β In Muscle Satellite Cells

**Figure 3:**
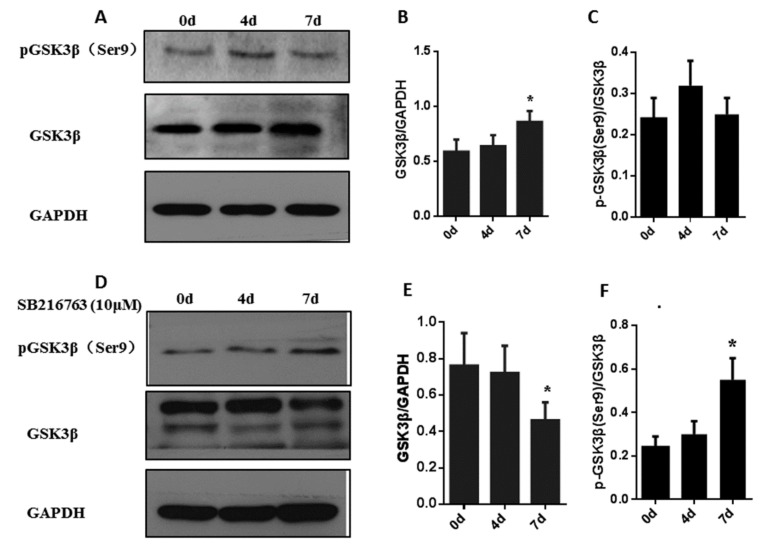
SB216763 affects the level of pGSK3β (Ser9) and GSK3β in muscle satellite cells. (**A**,**B**,**C**) Representative western blots and quantified results of pGSK3β (Ser9) and GSK3β during adipogenic differentiation in muscle satellite cells for 0, 4 and 7 days. (**D**,**E**,**F**) Representative western blots and quantified results of the levels of pGSK3β (Ser9) and GSK3β in muscle satellite cells treated with SB216763 for 0, 4 and 7 days. Error bars represent the SEMs of three separate experiments. ** *p* < 0.01; * *p* < 0.05 relative to 0 d level.

Without SB216763 treatment, the total protein level of GSK3β was significantly (*p* < 0.05) increased at 7 days after the adipogenic differentiation process of muscle satellite cells. In addition, there was no significant change in the level of pGSK3β (Ser9) during adipogenic differentiation (Figure 3A–C). To determine whether SB216763 reduces GSK3β activation, we evaluated pGSK3β (Ser9) and total GSK3β protein expression in muscle cells by western blot. The level of pGSK3β (Ser9) was significantly (*p* < 0.05) upregulated from 0 to 7 days, reaching a peak at 7 days. In addition, the total protein level of GSK3β was significantly (*p* < 0.05) decreased in response to SB216763 treatment at 7 days (Figure 3D–F). The results demonstrate that SB216763 reduces GSK3β activation by increasing the level of pGSK3β (Ser9) and decreasing the level of total GSK3β protein. 

### 3.4. SB216763 Inhibits Lipid Accumulation in Muscle Satellite Cells

**Figure 4:**
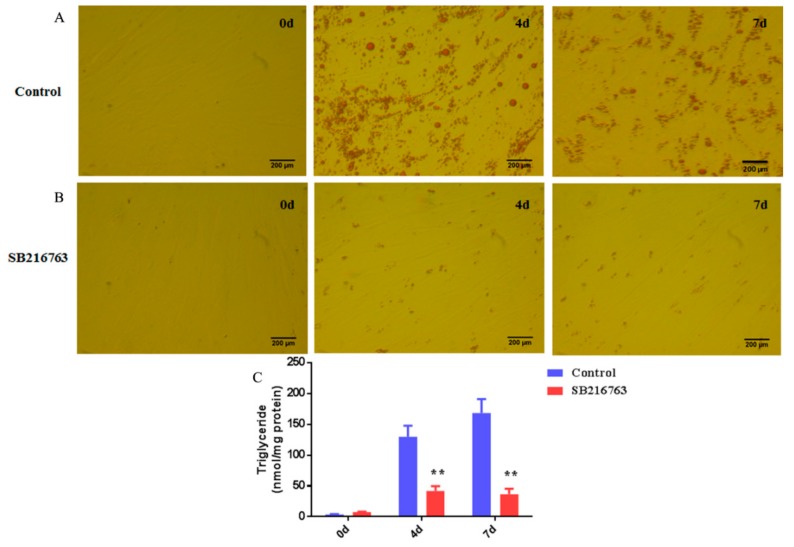
Inhibition of GSK3β decreased lipid accumulation in muscle satellite cells. (**A**,**B**) Cells were fixed and stained with Oil Red O on day 0, day 4 and day 7 with or without SB216763 treatment for 4 and 7 days. (**C**) Quantitative analysis of triglyceride (TG) content after the SB216763 treatment during the adipogenic differentiation. Error bars represent the SEMs of three separate experiments. ** *p* < 0.01 relative to TG content of without SB216763 treatment.

To determine the role of GSK3β in the lipid accumulation of muscle satellite cells; cells were treated with 10 μM SB216763 for 4 and 7 days to inhibit GSK3β activity. As shown in Figure 4A,B, SB216763 strongly inhibited lipid accumulation in muscle satellite cells compared with the control group. There was smaller number of lipid droplets in the SB216763 treatment group than that in the control group, indicating that GSK3β inhibition decreased lipid accumulation in muscle satellite cells. Furthermore, quantitative analysis of the triglyceride (TG) content revealed that SB216763 significantly (*p* < 0.01) decreased the TG content at 4 and 7 days compared with the control after the adipogenic differentiation of muscle satellite cells (Figure 4C). There was no obvious difference between 4 and 7 days after SB216763 treatment. These results suggest that GSK3β inhibition decreases the lipid accumulation of skeletal muscle satellite cells.

### 3.5. GSK3β Regulates the Expression of Lipogenesis-Related Genes in the Adipogenic Differentiation of Muscle Satellite Cells

**Figure 5:**
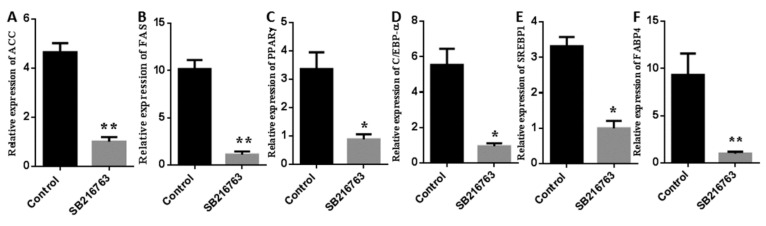
GSK3β regulates the expression of lipogenesis-related genes in muscle satellite cells. Cells were incubated with SB216763 (10 μM) for 7 days after adipogenic differentiation. Expression levels of ACC (**A**), FAS (**B**), PPARγ (**C**), C/EBP-α (**D**), SREBP1(**E**), and FABP4 (**F**) was determined by qPCR. Error bars represent the SEMs of three separate experiments. ** *p* < 0.01; * *p* < 0.05 relative to control level.

Then, we analyzed genes involved with lipogenesis-related genes after SB216763 treatment for 7 days. SB216763 suppressed the mRNA levels of and *ACC* (acetyl-CoA carboxylase) and *FAS* (fatty acid synthetase) (*p* < 0.01), key enzymes mediating fatty acid synthesis (Figure 5A,B). Several transcription factors in adipogenesis, PPARγ (peroxisome proliferators-activated receptor γ), C/EBPα (CCAAT enhancer binding protein α) and SREBP1 (sterol-regulatory element binding protein 1) were also (*p* < 0.05) downregulated after SB216763 treatment (Figure 5C–E). In addition, SB216763 significantly (*p* < 0.01) decreased the mRNA expression level of *FABP4* (fatty acid binding protein 4) (Figure 5F), which plays an important role in fatty acid transport.

### 3.6. GSK3β Regulates the Lipid Accumulation in Muscle Satellite Cells through Activating the Ampk Pathway

**Figure 6:**
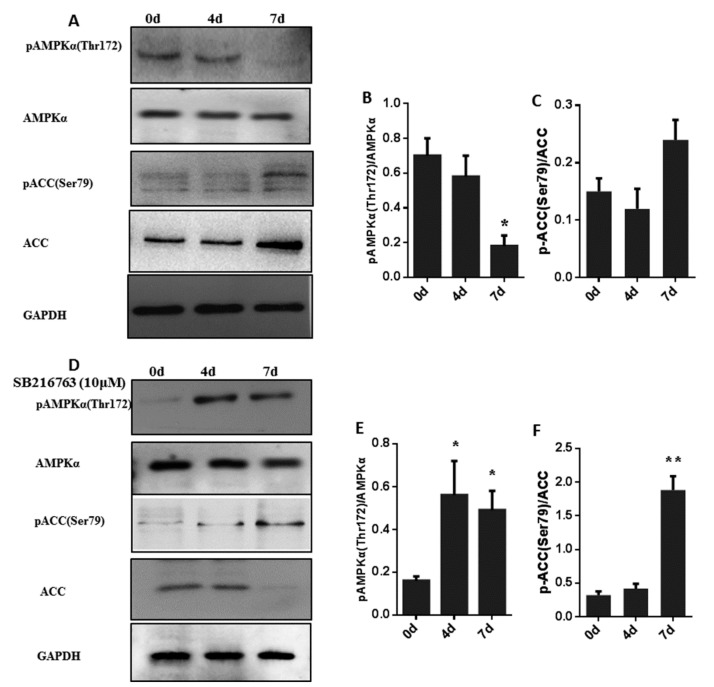
Inhibition of GSK3β increased the levels of pAMPKα (T172) and pACC (Ser79) in muscle satellite cells. (**A**,**B**,**C**) Representative western blots and quantified results of pAMPKα (T172) and pACC (Ser79) during adipogenic differentiation in muscle satellite cells for 0, 4 and 7 days. (**D**,**E**,**F**) Representative western blots and quantified results of pAMPKα (T172) and pACC (Ser79) in muscle satellite cells treated with SB216763 for 0, 4 and 7 days after adipogenic differentiation. Error bars represent the SEMs of three separate experiments. ** *p* < 0.01; * *p* < 0.05 relative to 0 d level.

To determine whether GSK3β regulates AMPK activation, we measured the phosphorylation levels of pAMPKα (T172) and pACC (Ser79) in muscle satellite cells with or without SB216763 treatment for 0, 4 and 7 days. As shown in Figure 6A–C, the level of pAMPKα (T172) was significantly (*p* < 0.05) decreased at 7 days after induction. There was no significant change in the level of pACC (Ser79) during adipogenic differentiation. After SB216763 treatment, the level of pAMPKα (T172) was significantly (*p* < 0.05) increased at 4 days after induction, and attained the highest level after 7 days. SB216763 did not induce any obvious alteration of total AMPKα protein (Figure 6D,E). In addition, as a direct substrate of AMPK, the level of pACC (Ser79) was significantly (*p* < 0.01) upregulated from 0 day to 7 days, reaching a peak at 7 days and the total ACC protein was significantly (*p* < 0.01) decreased in the 7 days of SB216763 stimulation (Figure 6D,F). 

**Figure 7:**
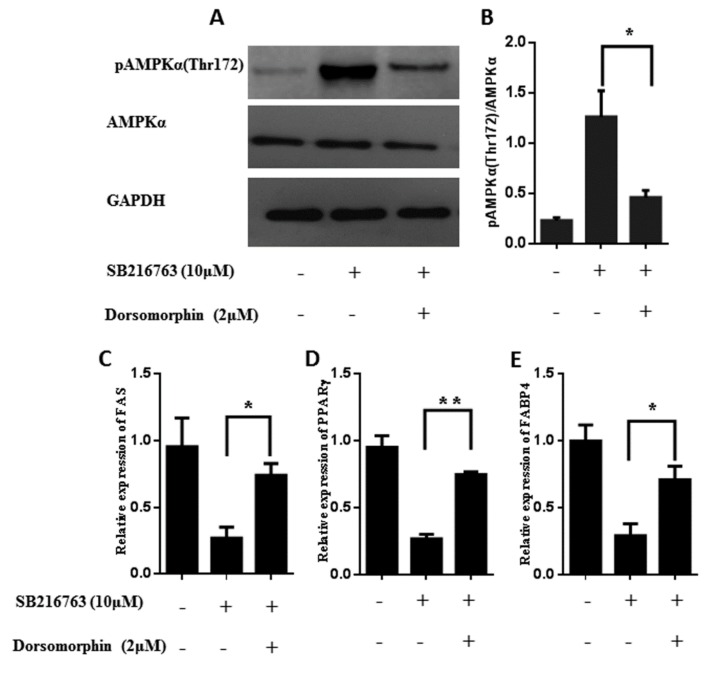
Inhibition of GSK3β reduced the lipid accumulation in muscle satellite cells through the AMPK pathway. (**A**,**B**) Representative western blots and quantified results of pAMPKα (T172) during adipogenic differentiation in muscle satellite cells for 4 days after SB216763/dorsomorphin treatment. (**C**,**D**,**E**) The expression levels of adipogenic-regulated genes (*FABP4*, *FAS* and *PPARγ*) during adipogenic differentiation for 4 days after SB216763/dorsomorphin treatment. Error bars represent the SEMs of three separate experiments. ** *p* < 0.01; * *p* < 0.05 relative to SB216763 treatment group.

Furthermore, we used an inhibitor of AMPK, dorsomorphin, to further confirm the role that GSK3β had in the lipid accumulation in muscle satellite cells through the AMPK pathway. As shown in Figure 7A,B, GSK3β inhibition increased the levels of pAMPKα (T172) and dorsomorphin attenuated the levels of pAMPKα (T172) (*p* < 0.01) induced by GSK3β inhibition. Then, we examined whether inactivation of AMPK could rescue the ectopic lipid deposition observed in the GSK3β inhibition. As expected, SB216763 reduced the lipid accumulation of muscle satellite cell and dorsomorphin treatment indeed increased the lipid accumulation compared with SB216763 treatment (Figure S1). In addition, the expression levels of adipogenic-regulated genes (*FABP4*, *FAS* and *PPARγ*) were significantly increased after SB216763 and dorsomorphin coincubation compared with SB216763 treatment (Figure 7C–E). These results demonstrate that GSK3β regulates the lipid accumulation in muscle satellite cells through activating the AMPK pathway. 

### 3.7. Inhibition of GSK3β-Induced Autophagy in Muscle Satellite Cells

**Figure 8:**
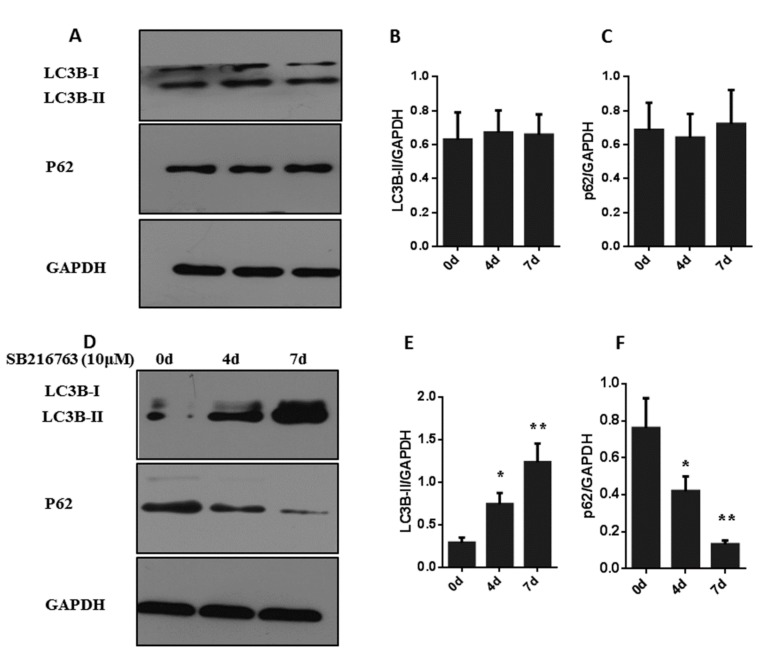
GSK3β inhibition induces autophagy in muscle satellite cells. (**A**,**B**,**C**) Representative western blots and quantified results of LC3B-II and p62 during adipogenic differentiation in muscle satellite cells for 0, 4 and 7 days. (**D**,**E**,**F**) Representative western blots and quantified results of LC3B-II and p62 in muscle satellite cells treated with SB216763 for 0, 4 and 7 days after adipogenic differentiation. Error bars represent the SEMs of three separate experiments. ** *p* < 0.01; * *p* < 0.05 relative to 0 d level.

The levels of the autophagy markers LC3B-II and p62 were assessed during adipogenic differentiation in muscle satellite cells. There were no changes in the levels of LC3B-II and p62 during adipogenic differentiation (Figure 8A–C). We, therefore, examined whether GSK3β inhibition regulates autophagy in muscle cells. The expressions of the autophagy markers LC3B-II and p62 were determined in muscle cells with SB216763 for 4 and 7 days to inhibit GSK3β activity. As shown in Figure 8D,E, the protein level of LC3B-II was weak at 0 day, but significantly (*p* < 0.05) decreased at 4 days after SB216763 treatment, reaching the highest expression level at 7 days (*p* < 0.01). The protein level of p62 was significantly (*p* < 0.01) downregulated in response to SB216763 treatment, reaching the lowest expression level at 7 days (Figure 8F). These data indicate that GSK3β inhibition promotes levels of LC3B-II and reduces the protein levels of p62 to induce the autophagy in the muscle satellite cells.

## 4. Discussion

Previous studies have demonstrated that GSK3β plays a key regulatory role in the regulation of adipocyte differentiation. After treatment with lithium chloride, an inhibitor of GSK3β, adipogenic differentiation was inhibited in human adipose-derived stem cells [21]. GSK3β inhibition reduced osteogenic differentiation mediated by the downregulation of β-catenin in mouse adipose-derived stromal cells [22]. Inhibition of GSK3β can increase the activity of glycogen synthase and glucose transport in 3T3-L1 adipocytes [23]. In addition, GSK3β inhibition prevents adipogenic differentiation and downregulated *PPARγ* and *C/EBPα* genes’ expressions. GSK3 promotes the binding of STAT5 to the promoter of the *secreted frizzled-related proteins* (*SFRPs*) gene to modulate adipocyte differentiation [24]. In this study, inhibition of GSK3β reduced lipid accumulation and decreased the expression of lipogenesis-related genes in the adipogenic differentiation of muscle satellite cells, suggesting that GSK3β plays important roles in ectopic lipid accumulation in muscle satellite cells.

Adipogenesis is a complex process of lipid biosynthesis controlled by the expression of lipogenesis related genes (*ACC* and *FAS*) and transcription factors (*SREBP1, PPARγ* and *C/EBPα*) [25,26]. It has been reported that *PPARγ* plays important roles in the regulation of target genes’ transcriptions during adipogenesis [27]. In addition, GSK3β activity is an essential regulatory factor during adipogenesis and promotes the expression of *PPARγ*, regulating the lipid differentiation in adipocytes [28]. *C/EBPα* overexpression upregulates *PPARγ* expression and induces the formation of lipid droplets in adipocytes during adipogenic differentiation [29]. Inhibition of GSK3β induces *C/EBPα* gene expression and promotes the ROS production in the spleens of zebrafish [30]. In goat mammary cells, overexpression of *SREBP1* increases the expression of lipogenesis-related genes and promotes triacylglycerol accumulation [31]. In this study, GSK3β significantly inhibited *SREBP1, PPARγ* and *C/EBPα* genes’ expressions, indicating that GSK3β is involved in regulating these transcription factors in response to inhibiting adipogenesis in muscle satellite cells.

AMPK has been proven to be one of substrates for GSK3β, which interacts with the AMPK heterotrimeric complex to regulate AMPK activity [32]. Inhibition of GSK3β activates AMPKα by direct phosphorylation at Thr172 to promote liver-innate immune activation [33]. We found that inhibition of GSK3β increased the phosphorylation levels of pAMPKα (T172) in muscle satellite cells. AMPK plays an important role in the regulation of lipid and glucose metabolism. AMPK suppresses lipid synthesis and promotes fatty acid oxidation by phosphorylating several substrates, including ACC (acetyl-CoA carboxylase), HSL (hormone-sensitive lipase) and ATGL (adipocyte-triglyceride lipase) [34,35]. Here, we found that SB216763 significantly increased the phosphorylation levels of pAMPKα (Thr172) and pACC (Ser79), respectively. In addition, SB216763 significantly decreased the lipid accumulation compared with the control. These results demonstrate that GSK3β affects lipid accumulation through AMPKα-ACC pathways in muscle satellite cells. 

The role of GSK3β in the regulation of autophagy is an understudied area, and it has been reported to modulate autophagy activity in several human diseases. In prostate cancer cells, inhibition of GSK3β promotes autophagy activity by the LKB1-AMPK pathway, in parallel with an increased protein level of LC-3B, and p62 protein reduction [36]. In an acute liver failure (ALF) mice model, GSK3β inhibition promotes autophagy to inhibit liver inflammation, indicating that GSK3β is involved in the hepatoprotective mechanisms though autophagic pathways [37]. GSK3β knockdown by shRNA interference hinders generation of malignancy and enhances autophagy though the AMPK pathway in breast cancer [38]. In this study, we found that GSK3β inhibition reduced the protein levels of p62 and promoted LC3II conversion to promote autophagy in the muscle cells. Taken together, our results provide new insight intothe critical function of autophagy in modulating lipid accumulation in muscle cells by the GSK3β-AMPK pathway.

## Supplementary Materials

The following are available online at https://www.mdpi.com/2073-4409/8/11/1378/s1. Table S1: Primer sequences used in this study. Figure S1: Inhibition of AMPK by Dorsomorphin can rescue the ectopic lipid deposition observed in muscle satellite cells. (**A**,**B**) Cells were fixed and stained with Oil Red O with SB216763/Dorsomorphin treatment for 0 and 4 days after adipogenic differentiation. (**C**) Quantitative analysis of TG content after the SB216763/Dorsomorphin treatment. Error bars represent the SEMs of three separate experiments. ** *p* < 0.01; * *p* < 0.05 relative to SB216763 treatment group.
