# Supplementary material for: Inhibition of GSK3β Reduces Ectopic Lipid Accumulation and Induces Autophagy by The AMPK Pathway in Goat Muscle Satellite Cells
Source: Cells. 2019 Nov 1;8(11):1378. doi: 10.3390/cells8111378 (PMC6912237; doi:10.3390/cells8111378)
Supplement: Supplementary file 1 [file cells-08-01378-s001.pdf]

Supplementary Materials

Table S1. Primer sequences used in this study.

Gene name	Primer name	Primer sequence (5'-3')	Size (bp)	Tm (ºC)
ACC	ACC-F<br>ACC-R	CCGTCTGTGATGACTTTGA<br>CTTTCTGGGTTGGGTGAG	191	57
FASN	FASN-F<br>FASN-R	CTGCTCAGTGGGCTCCTCA<br>TGGCGGTCAGTGGCTATGT	187	60.4
SREBP-1	SREBP1-F<br>SREBP1-R	CTGCTGACCGACATAGAAGACAT<br>GTAGGGCGGGTCAAACAGG	81	59.6
C/EBPα	CEBP-F<br>CEBP-R	GGCAACGACTTTGACTACCCG<br>TGCTTCGCTTCGTCCTCC	208	62.5
PPARγ	PPAR1-F<br>PPAR1-R	GTGTCACTCCTGAACGAAAT<br>GGAAATGCTGGAGAAGTCAA	156	60
FABP4	FABP4-F<br>FABP4-R	ACTGGGATGGGAAATCAACC<br>CCTTGGCTTATGCTCTCTCG	117	59
PPIA	PPIA-F<br>PPIA-R	AAGTCCCGAAGACAGCAGAA<br>GATGCCAGGACCTGTATGCT	209	60

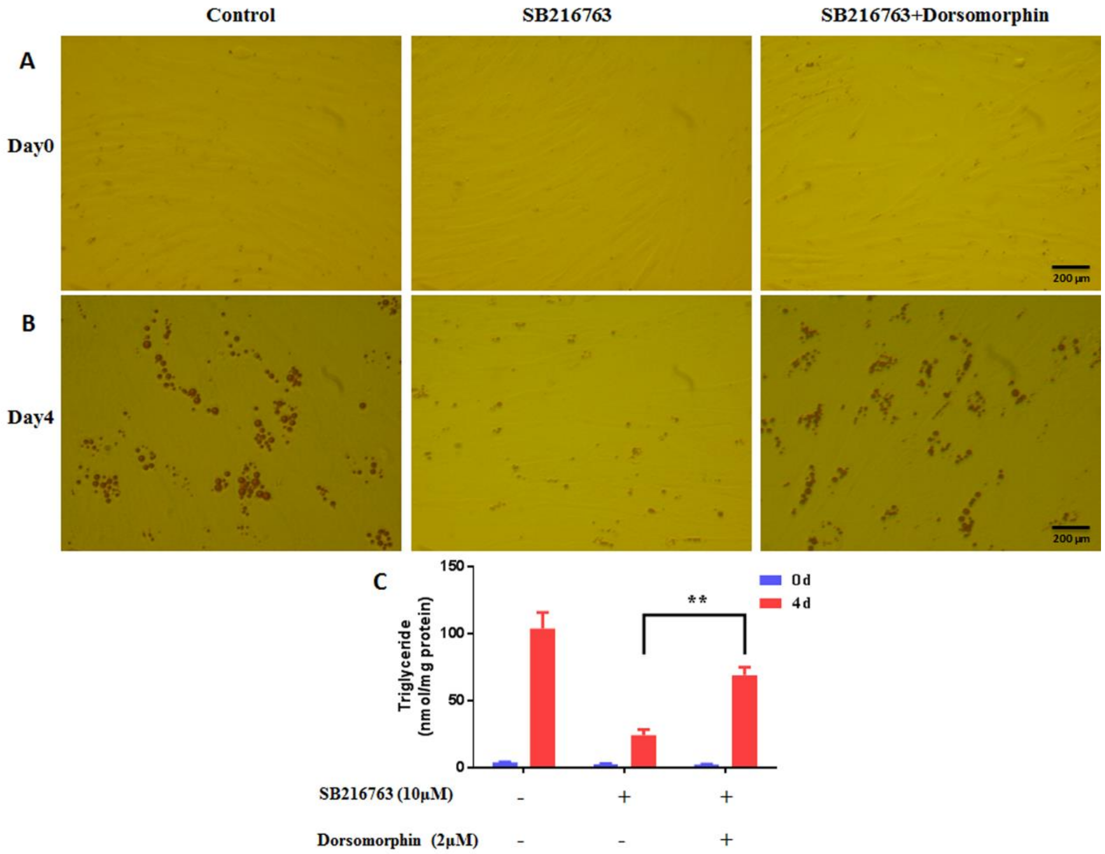

Figure S1. Inhibition of AMPK by Dorsomorphin can rescue the ectopic lipid deposition observed in muscle satellite cells. (A,B) Cells were fixed and stained with Oil Red O with SB216763/Dorsomorphin treatment for 0 and 4 days after adipogenic differentiation. (C) Quantitative analysis of TG content after the SB216763/Dorsomorphin treatment. Error bars represent the SEMs of three separate experiments. **p < 0.01; *p < 0.05 relative to SB216763 treatment group.
